# Supplementary material for: Chiral singlet superconductivity in the weakly correlated metal LaPt3P
Source: Nat Commun. 2021 May 4;12:2504. doi: 10.1038/s41467-021-22807-8 (PMC8097077; doi:10.1038/s41467-021-22807-8)
Supplement: Supplementary file 1 — Supplementary Information [file 41467_2021_22807_MOESM1_ESM.pdf]

Supplementary material to
"Chiral singlet superconductivity in the weakly correlated metal $LaPt_3P$"

P. K. Biswas,[1,*] S. K. Ghosh,[2,†] J. Z. Zhao,[3] D. A. Mayoh,[4] N. D. Zhigadlo,[5,6]
Xiaofeng Xu,[7] C. Baines,[8] A. D. Hillier,[1] G. Balakrishnan,[4] and M. R. Lees[4]

[1] *ISIS Pulsed Neutron and Muon Source, STFC Rutherford Appleton Laboratory, Harwell Campus, Didcot, Oxfordshire OX11 0QX, United Kingdom*
[2] *School of Physical Sciences, University of Kent, Canterbury CT2 7NH, United Kingdom*
[3] *Co-Innovation Center for New Energetic Materials, Southwest University of Science and Technology, Mianyang, 621010, China*
[4] *Physics Department, University of Warwick, Coventry, CV4 7AL, United Kingdom*
[5] *Laboratory for Solid State Physics, ETH Zurich, 8093 Zurich, Switzerland*
[6] *CrystMat Company, 8037 Zurich, Switzerland*
[7] *Department of Applied Physics, Zhejiang University of Technology, Hangzhou 310023,China*
[8] *Laboratory for Muon Spin Spectroscopy, Paul Scherrer Institute, CH-5232 Villigen PSI, Switzerland*

(Dated: March 30, 2021)

In this Supplemental Note, we present details of the synthesis, characterization measurements, experimental methods and data analysis of the $LaPt_3P$ samples grown at Warwick, United Kingdom and at ETH, Switzerland. We also give additional band structure results, details of the symmetry analysis and topological properties of the chiral d-wave state of $LaPt_3P$.

Supplementary Note 1. Synthesis and characterization of the sample grown at Warwick, United Kingdom

Polycrystalline $LaPt_3P$ [1] samples (sample-A) were synthesized by a solid state reaction method. Powders of elemental platinum, red phosphorus, and alkaline earth (lanthanum) were mixed in an argon-filled glove box, and sealed in a quartz tube filled with argon gas. The tube was initially heated to 400 °C and held at this temperature for 12 h in order to avoid rapid volatilization of phosphorus, then reacted at 900 °C for 72 h. The sintered pellet was reground and further annealed at 900 °C within argon-filled quartz tubes for several days and finally quenched into iced water.

The room-temperature structure was determined via powder x-ray diffraction (PXRD). PXRD was measured using a Bruker D5000 general purposed powder diffractometer. The diffraction pattern is shown in Supplementary Fig. 1. Reitveld refinement was carried out using the TOPAS software package [2] which gave the parameters shown in Supplementary Table 1.

Supplementary Table 1. Crystallographic and Rietveld refinement parameters obtained on $LaPt_3P$.

Space-group	*P4nmm* (No. 129)
Formula units/unit cell (Z)	2
Lattice parameter	
a(Å)	5.7683(6) (at 300K)
c(Å)	5.4681(7) (at 300K)
V_{cell}(Å^3)	182.4

Atom	Wyckoff Position	Occupancy	x	y	z
Pt(1)	4e	1	0.25	0.25	0.5
Pt(2)	2c	1	0	0.5	0.1476(8)
La(1)	2a	1	0	0.5	0.758(4)
P(1)	2c	1	0	0	0

The heat capacity in zero field was measured using a Quantum Design Physical Property Measurement System (PPMS) with a He^3 insert to get down to 0.5 K. The total specific heat C_{tot} at low temperatures is made up of several contributions,

$$C_{\rm tot} = C_{\rm el} + C_{\rm ph} + C_{\rm hyp} \tag{1}$$

where $C_{\rm el}$ is the electronic specific heat having the form in the normal state

$$C_{\rm el} = \gamma_n T \tag{2}$$

* pabitra.biswas@stfc.ac.uk
† S.Ghosh@kent.ac.uk

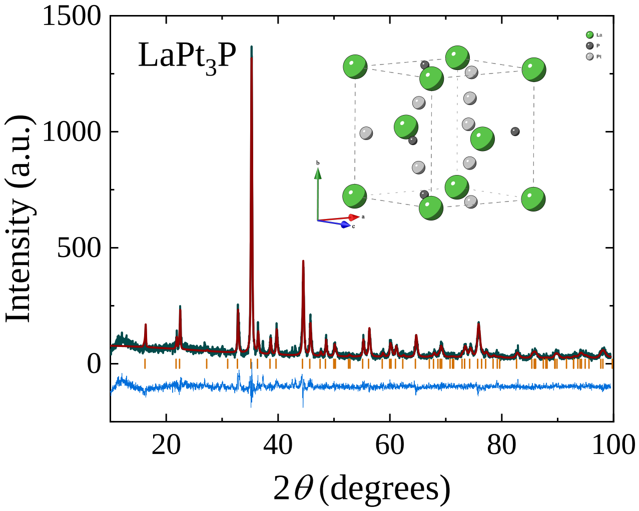

Supplementary Fig. 1. **Powder x-ray diffraction pattern of the sample-A of LaPt$_3$P at room temperature.** X-ray diffraction pattern of LaPt$_3$P at room temperature where the green, red and blue lines indicate the experimental data, the fit and the difference between the data and the fit, respectively. The orange dashes indicate the expected Bragg peaks. The inset shows the structure of a unit cell of LaPt$_3$P.

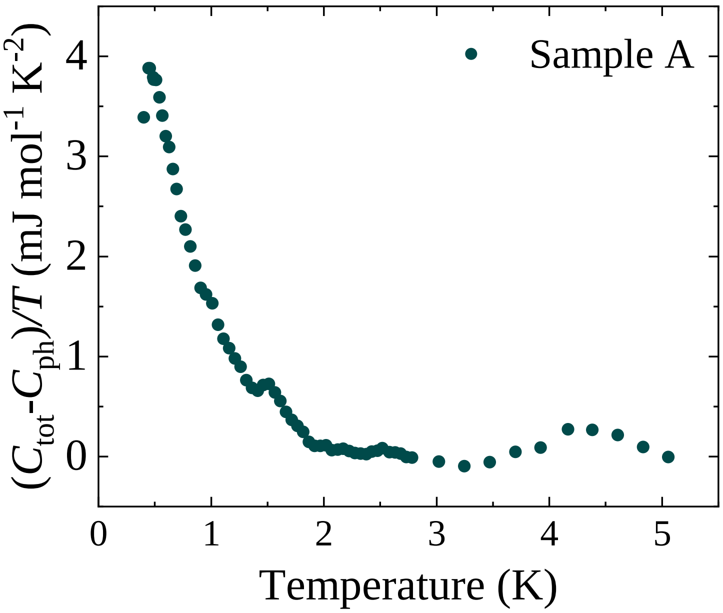

Supplementary Fig. 2. **Heat capacity of the sample-A of LaPt$_3$P in zero-field.** $(C_{tot} - C_{ph})/T$ as a function of temperature. There is a small anomaly close to the expected superconducting transition temperature that is masked by a large hyperfine contribution.

with γ_n being the Sommerfeld coefficient, C_{ph} is the specific heat due to the phonons given by

$$C_{\mathrm{ph}} = \beta_3 T^3 + \beta_5 T^5 \tag{3}$$

with β_3 and β_5 being temperature independent parameters, and C_{hyp} is a contribution due to hyerfine splitting

$$C_{\mathrm{hyp}} \propto 1/T^2. \tag{4}$$

Fitting the normal state specific heat gives $\gamma_n = 9.78(7)$ mJ/mol-K, $\beta_3 = 0.369(14)$ mJ/mol-K^4 and $\beta_5 = 5.47(5)$

μJ/mol-K^4. We then subtract the phonon contribution to the specific heat to plot the electronic specific heat including the hyperfine contribution. This is shown in the Supplementary Fig. 2 for the sample-A of $LaPt_3P$, and is consistent with the previous measurement of Ref.[1]. We note that the specific heat has a small anomaly close to the expected $T_c \approx 1.1$ K which is obscured by an upturn at lower temperatures. This is due to a large hyperfine contribution to the specific heat. We also note that there is an anomaly in the specific heat at $T \approx 1.5$ K which was also reported in Ref.[1]. The origin of this anomaly, however, is not clear and will be investigated in future experiments.

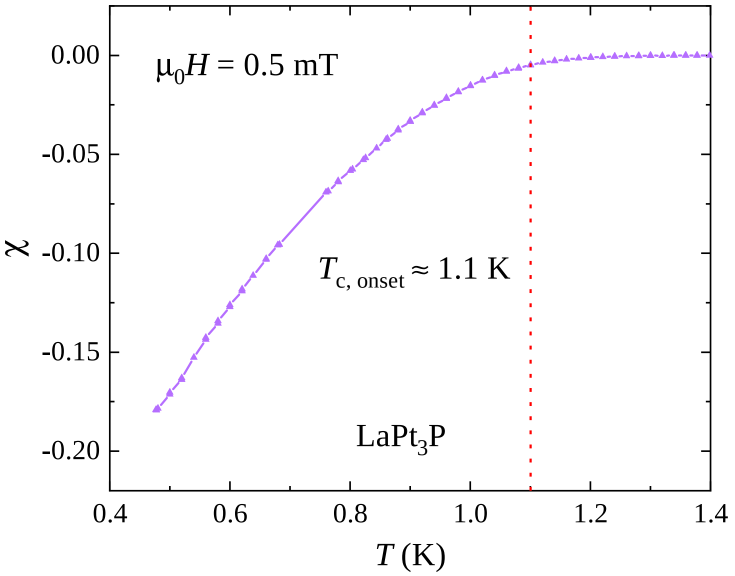

Supplementary Fig. 3. **Zero-field-cooled magnetic susceptibility of the sample-A of $LaPt_3P$ as a function of temperature.**

The magnetic susceptibility was measured using a Quantum Design Magnetic Property Measurement System (MPMS) using an i-quantum ^{3}He insert. As seen from Supplementary Fig. 3 this sample has a relatively low Meissner fraction ($\sim$ 30%).

Supplementary Note 2. Synthesis and characterization of the sample grown at ETH, Switzerland

A polycrystalline sample of $LaPt_3P$ (sample-B) was synthesized using the cubic anvil high-pressure and high-temperature technique. Starting powders of LaP and Pt of high purity (99.99%) were weighed according to the stoichiometric ratio, thoroughly ground, and enclosed in a boron nitride container, which was placed inside a pyrophyllite cube with a graphite heater. The details of experimental setup can be found in Ref.[3]. All the work related to the sample preparation and the packing of the high pressure cell-assembly was performed in an argon-filled glove box. In a typical run, a pressure of 2 GPa was applied at room temperature. The temperature was ramped in 3 h to the maximum value of 1500 °C, maintained for 5 h, and then cooled to 1350 °C over 5 h and finally reduced to room temperature in 3 h. Afterward, the pressure was released, and the sample was removed. The sample exhibits a large diamagnetic response with the superconducting transition temperature of 1.1 K.

Susceptibility measurements were performed using a Quantum Design Magnetic Property Measurement System (MPMS) by cooling the sample at base temperature in zero field and then apply 7 mT magnetic field. Data were collected while warming up the sample temperature. As shown in the main text, the temperature dependence of the susceptibility data shows a bulk superconducting transition with a T_c at around 1.1 K.

A virgin magnetisation curve was measured at 0.63 K in a Quantum Design MPMS. A linear deviation of the magnetisation curve at low field region (see Supplementary Fig. 4) shows that the lower critical field H_{c1} of $LaPt_3P$ is around 1 mT.

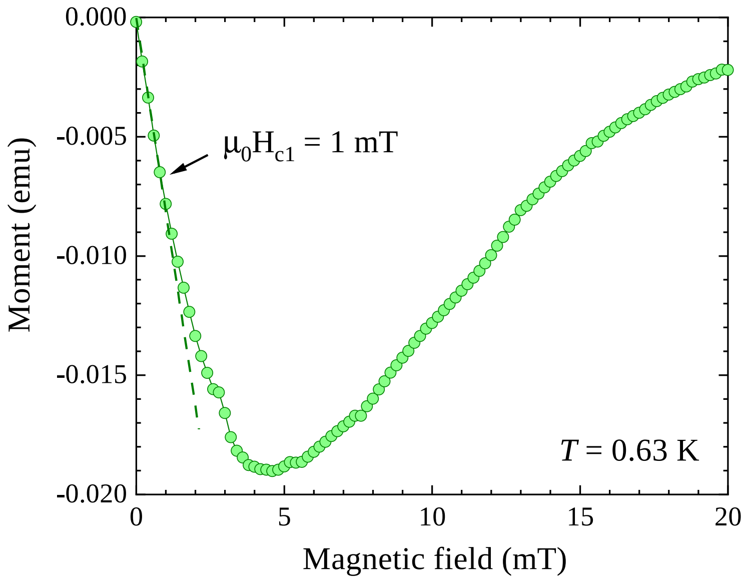

Supplementary Fig. 4. **Magnetic field dependence of the virgin magnetisation curve for sample-B of LaPt$_3$P.** We note that the lower critical field $\mu_0 H_{c1} \approx 1$ mT.

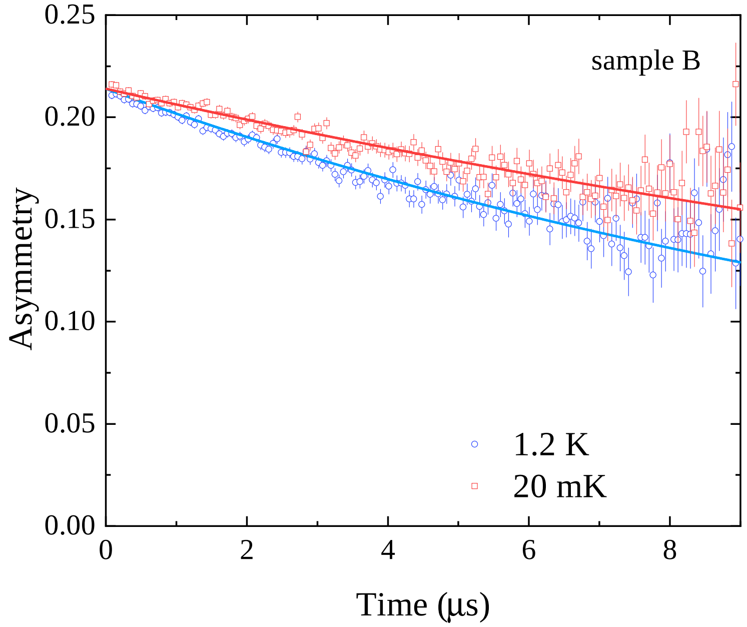

Supplementary Fig. 5. **ZF-μSR time spectra for sample-B of LaPt$_3$P.** The data collected at the two temperature values 20 mK and 1.2 K are shown.

Supplementary Note 3. μSR technique

μSR is a very sensitive local magnetic probe utilizing fully spin-polarized muons [4]. In a μSR experiment polarized muons are implanted into the host sample. After thermalization, each implanted muon decays (lifetime $\tau_\mu = 2.2$ μs) into a positron (and two neutrinos) emitted preferentially in the direction of the muon's spin at the time of decay. Using detectors appropriately positioned around the sample, the decay positrons are detected and time stamped. From the collected histograms, the asymmetry in the positron emission as a function of time, $A(t)$, can be determined, which is directly proportional to the time evolution of the muon spin polarization.

μSR measurements were performed on sample-A in the MUSR spectrometer at the ISIS Pulsed Neutron and

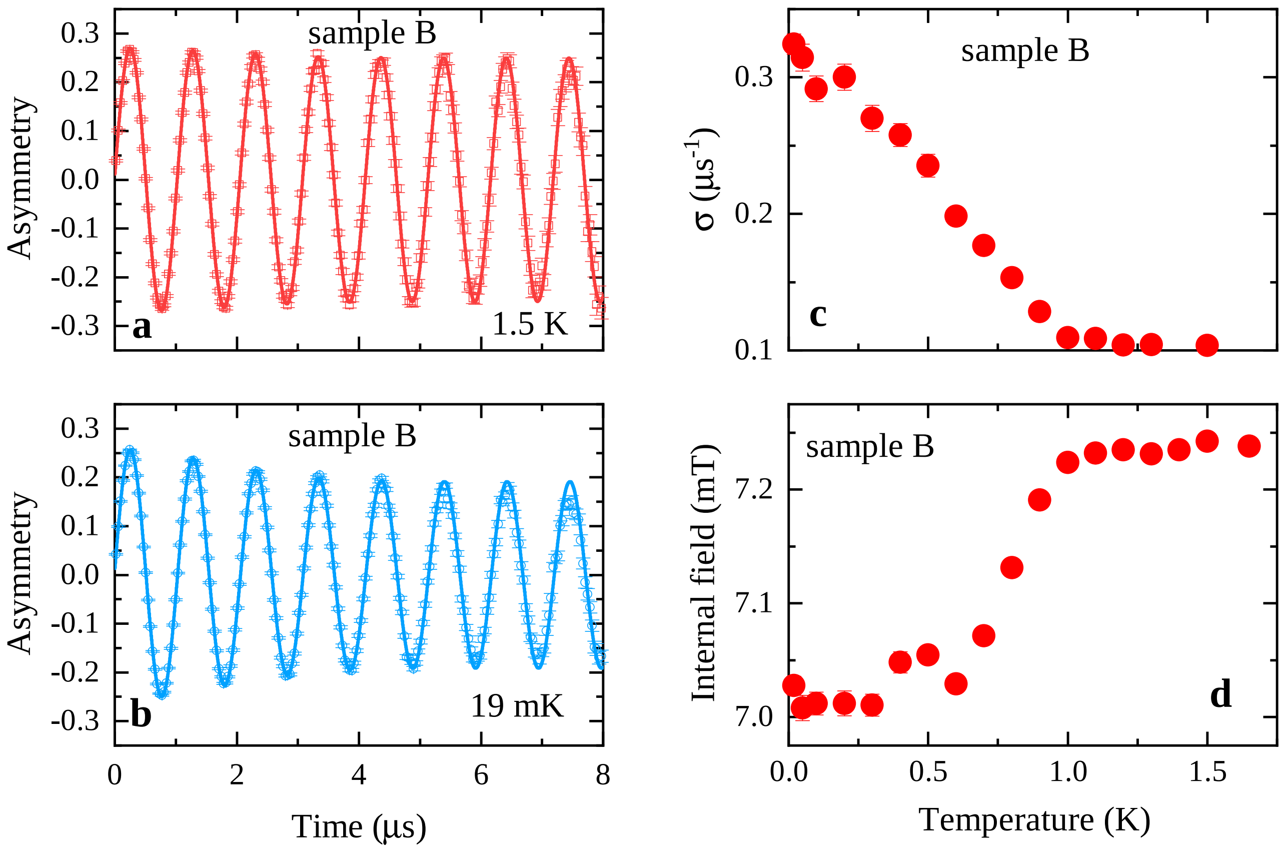

Supplementary Fig. 6. **TF-μSR time spectra for sample-B of $LaPt_3P$.** TF-μSR time spectra collected at a) 1.5 K and b) 19 mK in a transverse field of 7 mT. The solid lines are the fits to the data using Eq. 2 of the main text. The temperature dependence of the extracted c) σ and d) internal field.

Muon Source, UK, and on sample-B in the LTF spectrometer at the Paul Scherrer Institut (PSI), Switzerland. The polycrystalline samples of $LaPt_3P$ in the form of powder were mounted on high purity silver sample holders. The samples were cooled from above T_c to base temperature in zero field for ZF-μSR measurements, and in a field for the TF-μSR measurements. The external field was 10 mT for the TF-μSR measurements performed at ISIS and was 7 mT for the TF-μSR measurements performed at PSI. ZF-μSR measurements were performed in true zero field, achieved by three sets of orthogonal coils working as an active compensation system which cancel any stray fields at the sample position down to 1.0 μT. LF-μSR measurements were also performed under similar field-cooled conditions. The typical counting statistics were $\sim$ 40 and $\sim$ 24 million muon decays per data point at ISIS and PSI, respectively. The ZF-μSR time spectra for sample-B is shown in Supplementary Fig. 5 which is equivalent to that of sample-A shown in Fig. 1**a** of the main text. The TF-μSR time spectra for sample-B and corresponding σ and internal field are shown in Supplementary Fig. 6. This figure is equivalent to that of sample-A shown in Fig. 2 of the main text. The ZF-, LF- and TF-μSR data were analyzed using the equations given in the text.

The zero temperature upper critical field for $LaPt_3P$, $\mu_0 H_{c2} \approx 0.12$ T which is much larger than the applied transverse fields in the TF-μSR measurements. The detailed parameters for the analysis of superfluid density data from the TF-μSR measurements for the two samples using the different gap models mentioned in the main text are given in the Supplementary Table 2.

Supplementary Table 2. Summary of the analysis of the superfluid density data for the two samples of $LaPt_3P$.

Model	$g(\theta,\phi)$	Gap type	Reduced least-squared deviation (χ^2_r)	Fitted $\Delta_m(0)/(k_B T_c)$
s-wave	1	nodeless	13.025	1.270 ± 0.020
p-wave	$\sin(\theta)e^{i\phi}$	two point nodes	4.537	1.693 ± 0.029
chiral d-wave	$\sin(2\theta)e^{i\phi}$	two point nodes + a line node	2.238	1.989 ± 0.011

Supplementary Note 4. Band structure

$LaPt_3P$ crystallizes in a centrosymmetric primitive tetragonal crystal structure. The corresponding space group is P4/nmm (No. 129) which is nonsymmorphic. The point group of the Bravais lattice is D_{4h}. The nonsymmorphic symmetries within a unit cell include both screw axes and glide planes. We have performed detailed band structure calculations of $LaPt_3P$ using density functional theory (DFT). The corresponding band structure results with and without spin orbit coupling (SOC) are shown in Supplementary Fig. 7(a) and Supplementary Fig. 7(b) respectively. We note that this material has significant splitting of bands due to SOC [5]. The maximum band splitting caused by the SOC near the Fermi level is estimated to be $\sim$ 120 meV and is most apparent along the MX high symmetry direction. The SOC induced band splitting breaks the spin-symmetry and have important consequences in Cooper-pairing in this material.

The 3D Fermi surfaces were ploted by the `XCrySDen` packages [6]. The Fermi surfaces with SOC are shown in Supplementary Fig. 8. We note that there are four Fermi surfaces with the middle two shown in Supplementary Fig. 8(b) and Supplementary Fig. 8(c); and again in Supplementary Fig. 8(f) and Supplementary Fig. 8(g) from a different view, contributing the most to the density of states (DOS) at the Fermi level. This is seen from the projected DOS at the Fermi level shown in Supplementary Fig. 9. Supplementary Fig. 9(a) shows the contributions of the different atomic orbitals to the DOS at the Fermi level. We note that Pt-5d orbitals contribute the most. Thus $LaPt_3P$ is a multi-band system. Supplementary Fig. 9(b) shows the contributions of the different Fermi surfaces to the DOS at the Fermi level.

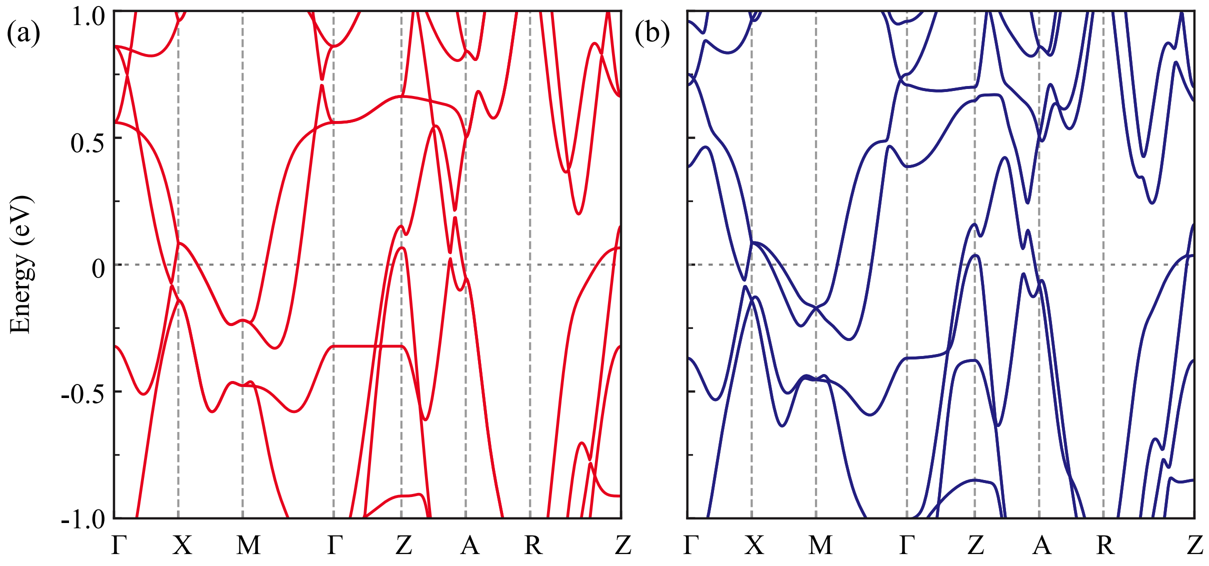

Supplementary Fig. 7. **First principles band structure results of $LaPt_3P$.** a) Band structure without SOC. b) Band structure with SOC. The high symmetry points and directions used in the band structure computation are for a standard primitive tetragonal Brillouin zone. We note that SOC induces significant band splitting near the Fermi level especially from M to X.

Supplementary Note 5. Symmetry analysis

In this section we describe, the symmetry analysis of the possible superconducting order parameters for $LaPt_3P$. To proceed, we note the properties of the material: it is centrosymmetric, has nonsymmorphic symmetries, has considerable effects of SOC, has multiple bands potentially participating in superconductivity, has spontaneously broken TRS at T_c and has line nodes dominating its thermodynamic behavior.

The normal state symmetry group of the system is given by $\mathcal{G} = G_0 \otimes U(1) \otimes \mathcal{T}$, where $U(1)$ is the gauge symmetry group, G_0 is the group of symmetries containing the point group symmetries of D_{4h} and spin rotation symmetries in 3D of $SO(3)$ and $\mathcal{T}$ is the group of time-reversal symmetry (TRS). The Ginzburg-Landau (GL) free energy of the system must be invariant under this symmetry group.

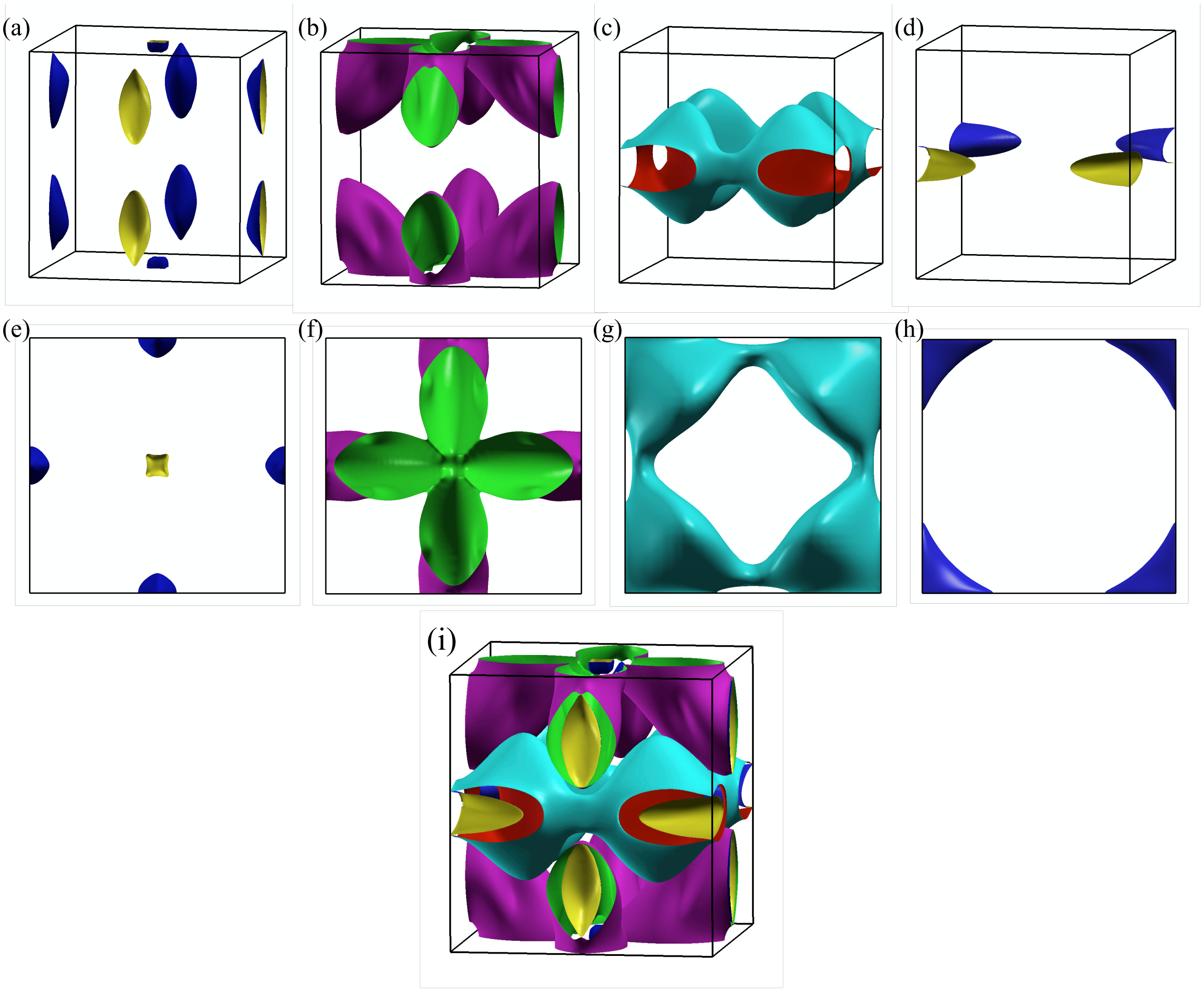

Supplementary Fig. 8. **Fermi surfaces of $LaPt_3P$ with SOC.** Panels (a)–(d) are from a side view and the panels (e)–(h) are from the top view for the four Fermi surface sheets and (i) shows a combined Fermi surface.

The D_{4h} point group has 8 one-dimensional irreducible representations (irreps) (4 of them have even parity and the other 4 have odd parity) and 2 two dimensional irreps (one with even parity denoted by E_g and the other with odd parity denoted by E_u). Centrosymmetry implies that this material has either purely triplet or purely singlet superconducting instability in general. Furthermore, a TRS breaking superconducting order parameter requires degenerate or multi-dimensional irreps. This system can thus lead to such type of instability only in the E_g or the E_u irrep. We will now focus only on these two irreps and construct possible superconducting order parameters for the system. We consider strong SOC as uncovered by the band structure calculation of this material.

The fourth order invariant corresponding to the 2 two-dimensional irreps E_g and E_u of D_{4h} gives the quartic order term of the GL free energy [7, 8] to be

$$f_4 = \beta_1(|\eta_1|^2 + |\eta_2|^2)^2 + \beta_2|\eta_1^2 + \eta_2^2|^2 + \beta_3(|\eta_1|^4 + |\eta_2|^4) \tag{5}$$

where (η_1, η_2) are the two complex components of the two-dimensional order parameters. This free energy needs to be minimized with respect to both η_1 and η_2. The non-equivalent solutions are: $(\eta_1, \eta_2) = (1, 0)$, $\frac{1}{\sqrt{2}}(1, 1)$ and $\frac{1}{\sqrt{2}}(1, i)$. There is an extended region in the parameter space where the states corresponding to $(\eta_1, \eta_2) = (1, i)$ is stabilized. The instabilities corresponding to this case spontaneously break TRS at T_c due to a non-trivial phase difference between the two order parameter components.

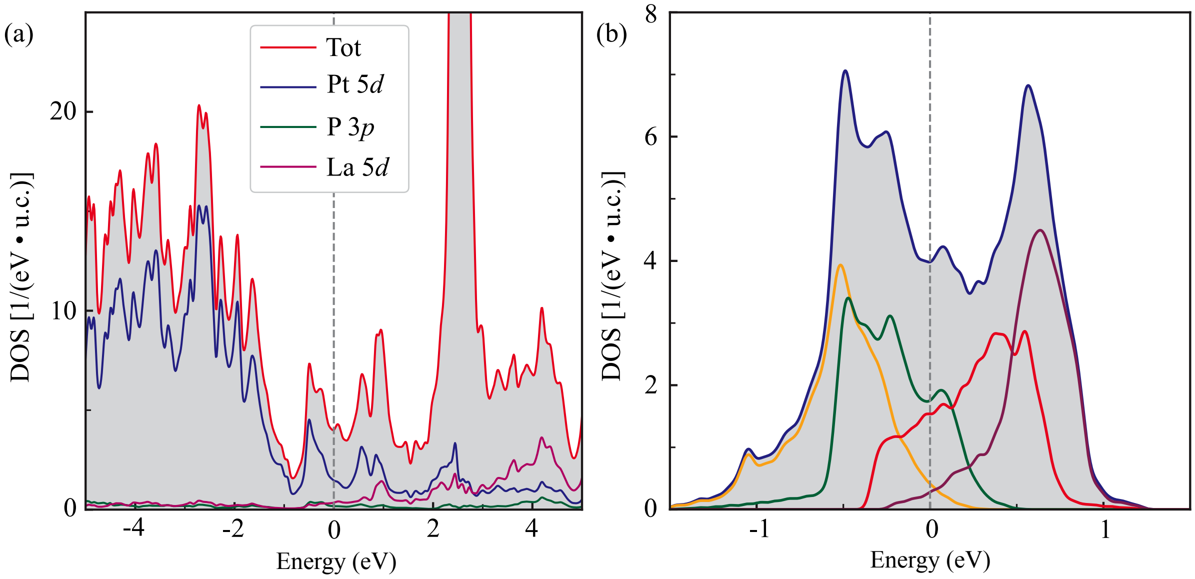

Supplementary Fig. 9. **Projected density of states (DOS) results.** Left panel shows the contributions of different orbitals to the DOS. We note that Pt 5d orbitals contribute the most to the DOS at the Fermi level. The right panel shows the DOS contributions of the different Fermi surfaces. The blue is total and the other four correspond to the four Fermi surfaces. Their contributions at the Fermi level are 10.3%, 43.4%, 39.5% and 6.3%.

Then the even parity superconducting order parameter belonging to E_g is given by

$$\Delta(\mathbf{k}) = \Delta_0 \mathrm{k_z}(\mathrm{k_x} + \mathrm{i}\mathrm{k_y}) \tag{6}$$

where Δ_0 is the real amplitude independent of $\mathbf{k}$. This is a *chiral d-wave* singlet order parameter. The odd parity superconducting order parameter belonging to E_u gives rise to the gap matrix $\hat{\Delta}(\mathbf{k}) = [\mathbf{d}(\mathbf{k}).\tilde{\sigma}]\mathrm{i}\sigma_\mathrm{y}$ where $\vec{\sigma}$ denotes the three Pauli spin matrices and $\mathbf{d}(\mathbf{k})$ is the triplet d-vector given by

$$\mathbf{d}(\mathbf{k}) = [\mathrm{Ak_z}, \mathrm{iAk_z}, \mathrm{B}(\mathrm{k_x} + \mathrm{ik_y})]\,. \tag{7}$$

Here, A and B are material dependent real constants independent of $\mathbf{k}$ and in general they are nonzero. We note that the values of A and B determine the orientation of the d-vector. For example, for $A = 0$ the d-vector points along the c-axis and for $B = 0$ the d-vector points in the ab-plane. We also note that

$$\mathbf{d}(\mathbf{k}) \times \mathbf{d}^*(\mathbf{k}) = 2\mathrm{iAk_z}(\mathrm{Bk_x}\hat{\mathrm{x}} - \mathrm{Bk_y}\hat{\mathrm{y}} - \mathrm{Ak_z}\hat{\mathrm{z}}) \tag{8}$$

which is nonzero in general. Hence, this superconducting state is *nonunitary chiral p-wave* triplet state.

The strong SOC case considered here implies that the single particle states are no longer the eigenstates of spin and we need to label them rather by pseudospins. The pseudospin states are linear combinations of the spin eigenstates. Since the pseudospin and the spin are closely related, the even parity states correspond to pseudospin singlet and the odd parity states correspond to pseudospin triplet states.

We can now follow the standard Bogoliubov-de Gennes mean field theory [8] to compute the quasi-particle excitation energy spectrum for the two TRS breaking states given in Supplementary Eq. (6) and Supplementary Eq. (7). The schematic view of the excitation energy gaps for the two order parameters are shown in Supplementary Fig. 10.

Supplementary Note 6. Topological properties of the chiral singlet state

To discuss the topological properties of the nodal excitations for the chiral d-wave state with the gap function

$$\Delta(\mathbf{k}) = \frac{\Delta_0}{\mathrm{k_F^2}} \mathrm{k_z}(\mathrm{k_x} + \mathrm{ik_y}) \tag{9}$$

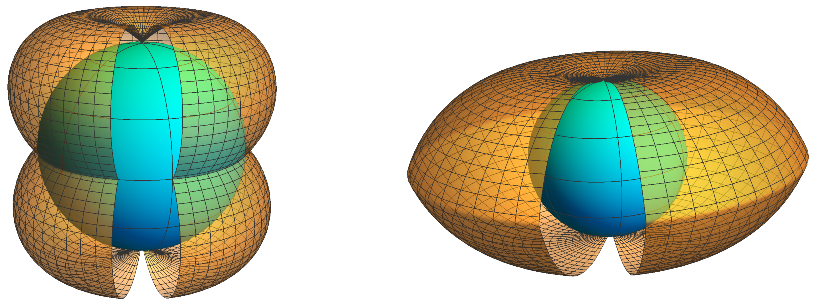

Supplementary Fig. 10. **Polar plots of the excitation energy gaps.** (a) The chiral d-wave singlet case and (b) the nonunitary chiral p-wave triplet case. In both the cases, point nodes appear at the two "poles", while the singlet case has an additional line node at the "equator".

with Δ_0 being the pairing amplitude, we assume a simplified single band parabolic dispersion (in units of $\hbar$)

$$\xi(\mathbf{k}) = \frac{\mathrm{k}^2}{2\mathrm{m}} - \mu, \tag{10}$$

where m is the mass of an electron, $\mu = \frac{k_F^2}{2m}$ is the chemical potential and k_F is the Fermi wavevector. We note that $\Delta(\mathbf{k}) \sim \mathrm{Y}_1^2(\theta, \phi)$ where $Y_m^l(\theta, \phi)$ are the spherical harmonics. Thus the Cooper pairs have an angular momentum $L_z = +1$ for this state.

Then Bogoliubov-de Gennes Hamiltonian in the pseudospin basis can be written as

$$\mathcal{H} = \sum_{\mathbf{k}} \Psi_{\mathbf{k}}^\dagger H(\mathbf{k}) \Psi_{\mathbf{k}} \tag{11}$$

where $\Psi_{\mathbf{k}} = (c_{\mathbf{k}\uparrow}, c_{-\mathbf{k}\uparrow}^\dagger)^T$ with $c_{\mathbf{k}\sigma}$ being the fermion annihilation operator with pseudospin flavor $\sigma \in \{\uparrow, \downarrow\}$. We can rewrite the BdG Hamiltonian as

$$H(\mathbf{k}) = \mathbf{N}(\mathbf{k}) \cdot \boldsymbol{\tau} \tag{12}$$

where $\boldsymbol{\tau}$ is the vector of the three Pauli matrices in the particle-hole space and $\mathbf{N}(\mathbf{k}) = \left\{ \frac{\Delta_0}{\mathrm{k_F^2}} \mathrm{k_z k_x}, \frac{\Delta_0}{\mathrm{k_F^2}} \mathrm{k_z k_y}, \xi(\mathbf{k}) \right\}$ is a pseudospin vector. The eigenvalues of the Hamiltonian in Supplementary Eq. (12) are $\pm E(\mathbf{k})$ where

$$E(\mathbf{k}) = |\mathbf{N}(\mathbf{k})| = \sqrt{\xi^2(\mathbf{k}) + |\Delta(\mathbf{k})|^2}. \tag{13}$$

Hence, the superconducting ground state has two point nodes at the two poles of the Fermi surface $\mathbf{k}_\pm = (0, 0, \pm \mathrm{k_F})$ and a line node at the equator $k_z = 0$ plane. The low energy Hamiltonian close to two point nodes can be written as

$$H(\mathbf{k}) = \frac{\Delta_0}{\mathrm{k_F}} (\mathrm{p_x} \tau_\mathrm{x} - \mathrm{p_y} \tau_\mathrm{y}) \pm \mathrm{v_F p_z} \tau_\mathrm{z} \tag{14}$$

where we have defined $\boldsymbol{p} = (\mathbf{k} - \mathbf{k}_\pm)$. This is a Weyl Hamiltonian. Thus the two point nodes are also Weyl nodes. As a result they are impossible to gap out since there is no fourth Pauli matrix which can come from a mass term to gap out the nodes.

The corresponding Bloch wave functions $|u_\pm(\mathbf{k})\rangle$ are the eigenfunctions of $\hat{\mathbf{n}}(\mathbf{k}).\boldsymbol{\sigma}$ with eigenvalues ± 1 where $\hat{\mathbf{n}}(\mathbf{k}) = \mathbf{N}(\mathbf{k})/|\mathbf{N}(\mathbf{k})|$ is the unit vector along the direction of the pseudospin $\mathbf{N}(\mathbf{k})$. We note that this unit vector $\hat{\mathbf{n}}(\mathbf{k})$ is well defined only when $|\mathbf{N}(\mathbf{k})| \neq 0$ i.e. in the nodeless regions on the Fermi surface. In spherical coordinates, parametrizing $\hat{\mathbf{n}}(\mathbf{k}) = [\mathrm{n_x}(\mathbf{k}), \mathrm{n_y}(\mathbf{k}), \mathrm{n_z}(\mathbf{k})] = [\sin(\theta)\cos(\phi), \sin(\theta)\sin(\phi), \cos(\theta)]$ we have

$$|u_-(\mathbf{k})\rangle = \begin{bmatrix} \cos(\frac{\theta}{2}) e^{-i\phi} \\ \sin(\frac{\theta}{2}) \end{bmatrix} \text{ and } |\mathrm{u}_+(\mathbf{k})\rangle = \begin{bmatrix} \sin(\frac{\theta}{2}) e^{-i\phi} \\ -\cos(\frac{\theta}{2}) \end{bmatrix}. \tag{15}$$

Then from the negative energy occupied states $|u_-(\mathbf{k})\rangle$ the Berry connection is defined as

$$\mathbf{A}(\mathbf{k}) = \mathrm{i} \langle \mathrm{u}_-(\mathbf{k}) | \boldsymbol{\nabla}_{\mathbf{k}} | \mathrm{u}_-(\mathbf{k}) \rangle \tag{16}$$

and the corresponding Berry curvature is $\mathbf{F}(\mathbf{k}) = \boldsymbol{\nabla}_{\mathbf{k}} \times \mathbf{A}(\mathbf{k})$. In terms of the components of $\hat{\mathbf{n}}(\mathbf{k})$, it is given by $\mathbf{F}(\mathbf{k}) = [\mathrm{n_y}(\mathbf{k})\{\boldsymbol{\nabla}_{\mathbf{k}}\mathrm{n_z}(\mathbf{k}) \times \boldsymbol{\nabla}_{\mathbf{k}}\mathrm{n_x}(\mathbf{k})\} - \mathrm{n_x}(\mathbf{k})\{\boldsymbol{\nabla}_{\mathbf{k}}\mathrm{n_z}(\mathbf{k}) \times \boldsymbol{\nabla}_{\mathbf{k}}\mathrm{n_y}(\mathbf{k})\}]/[2\{\mathrm{n_x^2}(\mathbf{k}) + \mathrm{n_y^2}(\mathbf{k})\}]$.

For the chiral d-wave case, $F_x(\mathbf{k})$ and $F_y(\mathbf{k})$ are odd functions of (k_y, k_z) and (k_x, k_z) respectively. Hence, there is no Berry flux along the x and y directions. The number of field lines coming in and out of the ca and cb planes are the same. Whereas $F_z(\mathbf{k})$ is an even function of (k_x, k_y) and the flux through the ab plane as a function of k_z is

$$\Phi(\mathbf{k}) = \int \mathrm{dk_x dk_y F_z}(\mathbf{k}) = 2\pi\mathcal{C}(\mathrm{k_z}). \tag{17}$$

$\mathcal{C}(k_z)$ is the "sliced" Chern number (momentum dependent) of the effective 2D problem for a fixed k_z. For a given value of $|k_z| < k_F$, the Hamiltonian in Supplementary Eq. (12) describes an effective 2D problem with fully gapped weak coupling BCS pairing and an effective chemical potential $\frac{\hbar^2}{2m}(k_F^2 - k_z^2)$ having the Chern number $C(k_z) = +1$. For $|k_z| > k_F$, the effective chemical potential is negative and describes a topologically trivial BEC state. Thus, the Weyl point nodes at $(0, 0, \pm k_F)$ act as monopoles and anti-monopoles of the Berry curvature and the flux through a sphere surrounding the monopole is 2π and that through the anti-monopole is -2π. The topologically protected Weyl nodes give rise to Majorana arc surface states on the surface Brillouin zone corresponding to the $(1, 0, 0)$ and $(0, 1, 0)$ surfaces having chiral linear dispersions along y and x directions respectively. As a result of the arc surface states the system shows anomalous thermal and spin Hall effects [9–11].

The equatorial line node is characterized by a 1D winding number. This can be defined in terms of the following spectral symmetry [10, 11] of the Hamiltonian. We note that the operator

$$\Gamma_{\mathbf{k}} = \sin(\phi_{\mathbf{k}})\tau_x + \cos(\phi_{\mathbf{k}})\tau_y \tag{18}$$

where $\tan(\phi_{\mathbf{k}}) = k_y/k_x$ anti-commutes with the Hamiltonian

$$\{H(\mathbf{k}), \Gamma_{\mathbf{k}}\} = 0. \tag{19}$$

As a result any eigenstate of the Hamiltonian $H(\mathbf{k})$ with the eigenvalue $E_{\mathbf{k}}$ is also an eigenstate of the operator $\Gamma_{\mathbf{k}}$. Then with the help of this spectral symmetry $\Gamma_{\mathbf{k}}$ we define the winding number as

$$w(\mathbf{k}_\perp) = -\frac{1}{4\pi i} \oint_{\mathcal{L}} \mathrm{d}l \; Tr \left[\Gamma_{\mathbf{k}} H^{-1}(\mathbf{k}) \partial_l \mathrm{H}(\mathbf{k})\right], \tag{20}$$

where dl is the line element along a closed loop $\mathcal{L}$ encircling the line node and $\mathbf{k}_\perp = (k_x, k_y)$. For this case then we have

$$w(\mathbf{k}_\perp) = 1 \quad \forall\, k_\perp < k_F \tag{21}$$
$$= 0 \quad \text{otherwise.} \tag{22}$$

We note that the winding number does not depend on the angular momentum of the Cooper pairs. This non-trivial topology of the line node ensures the existence of zero-energy surface Andreev bound states on the $(0, 0, 1)$ surface. They produce an image of the Fermi surface equator in the corresponding surface Brillouin zone. Being dispersionless, these zero-energy states result in a divergent density of states, and are predicted to give rise to a zero bias peak in tunnelling measurements. These zero modes are two fold degenerate Majorana fermions arising from the twofold spin degeneracy of the pairing interaction.

[1] Takayama, T. *et al.* Strong Coupling Superconductivity at 8.4 K in an Antiperovskite Phosphide $SrPt_3P$. *Phys. Rev. Lett.* **108**, 237001 (2012).

[2] Coelho, A. A. *TOPAS* and *TOPAS-Academic*: an optimization program integrating computer algebra and crystallographic objects written in C++. *J. Appl. Crystallogr.* **51**, 210–218 (2018).

[3] Zhigadlo, N. D. High pressure crystal growth of the antiperovskite centrosymmetric superconductor $SrPt_3P$. *J. Cryst. Growth* **455**, 94 – 98 (2016).

[4] Yaouanc, A. & de Réotier, P. D. *Muon Spin Rotation, Relaxation, and Resonance: Applications to Condensed Matter* (Oxford University Press, Oxford, 2011).

[5] Chen, H., Xu, X., Cao, C. & Dai, J. First-principles calculations of the electronic and phonon properties of $A\mathrm{Pt_3P}$ (A = Ca, Sr, and La): Evidence for a charge-density-wave instability and a soft phonon. *Phys. Rev. B* **86**, 125116 (2012).

[6] Kokalj, A. Computer graphics and graphical user interfaces as tools in simulations of matter at the atomic scale. *Comput. Mater. Sci.* **28**, 155–168 (2003).

[7] Annett, J. F. Symmetry of the order parameter for high-temperature superconductivity. *Adv. Phys.* **39**, 83–126 (1990).
[8] Sigrist, M. & Ueda, K. Phenomenological theory of unconventional superconductivity. *Rev. Mod. Phys.* **63**, 239–311 (1991).
[9] Schnyder, A. P. & Brydon, P. M. R. Topological surface states in nodal superconductors. *J. Phys. Condens. Matter* **27**, 243201 (2015).
[10] Goswami, P. & Nevidomskyy, A. H. Topological Weyl superconductor to diffusive thermal Hall metal crossover in the B-phase of UPt_3. *Phys. Rev. B* **92**, 214504 (2015).
[11] Goswami, P. & Balicas, L. Topological properties of possible Weyl superconducting states of URu_2Si_2. Preprint at https://arxiv.org/abs/1312.3632 (2013).
